# Treatment of infected predators under the influence of fear-induced refuge

**DOI:** 10.1038/s41598-023-43021-0

**Published:** 2023-10-03

**Authors:** Bapin Mondal, Abhijit Sarkar, Nazmul Sk

**Affiliations:** 1 Department of Applied Mathematics, University of Calcutta, Kolkata, 700009 India; 2 Department of Mathematics, JIS College of Engineering, Kalyani, Kalyani, 741235 India; 3 Department of Mathematics, University of Kalyani, Kalyani, 741235 India

**Keywords:** Ecology, Mathematics and computing

## Abstract

In this research, we delve into the dynamics of an infected predator–prey system in the presence of fear and refuge, presenting a novel inclusion of treatment for infected individuals in this type of model. Through our analytical efforts, we establish a significant reproduction number that holds a pivotal role in determining disease extinction or persistence within the system. A noteworthy threshold value for this reproduction number delineates a boundary below which the infected population cannot endure in the system. It’s important to note that a range of reproduction numbers leads to both disease-free and endemic scenarios, yet the stability of these situations is contingent upon the initial population sizes. Furthermore, our investigation extends to the exploration of various types of bifurcation-namely, Backward, Saddle-node, and Hopf bifurcations. These findings unravel the intricate and diverse dynamics of the system. Of particular significance is the derivation of an optimal control policy for treatment, augmenting the practical utility of our work. The robustness of our analytical findings is fortified through meticulous verification via numerical simulations. These simulations not only bolster the credibility of our analytical results but also enhance their accessibility. Our study unveils that fear, refuge, and treatment possess individual capabilities to eradicate the disease from the system. Notably, increasing levels of fear and refuge exert a passive influence on the elimination of the infected population, whereas treatment wields an active influence-a crucial insight that bolsters the foundation of our model. Furthermore, our investigation uncovers a spectrum of system dynamics including bistability, one-period, two-period, and multi-period/chaotic behavior. These discoveries contribute to a profound enrichment of the system’s dynamic landscape.

## Introduction

The study of population interactions through mathematical models has become an integral part of ecology. Over the last few decades, mathematical models have been extensively studied to explore the rich dynamics they offer. Among the most important mathematical population models, predator–prey models play a crucial role in understanding the dynamics of different species. These models are developed to capture volatile natural phenomena and have been extensively studied and widely investigated^1–5^. In nature, it’s common for predators to hunt prey. However, predators cannot capture all prey because the latter can exhibit anti-predator behaviors like seeking refuge, allowing them to evade predation^6–8^. This indicates that refuge behavior protects and prevents the prey from extinction, thus playing a pivotal role in the interaction between prey and predator populations. Recent research on prey refuges in prey–predator models has captured the attention of mathematicians, leading to the discovery of interesting dynamics^9–12^. Moreover, most predator–prey models assume direct predation as the only way prey are killed. However, this does not align with the real scenario, where prey alter their behavior in the presence of predators^13–15^. Recent studies have demonstrated that prey exhibit anti-predator behavior due to predation risks, including habitat changes, vigilance, foraging adjustments, and internal changes. While these anti-predator behaviors enhance short-term survival, they significantly impact reproduction costs in the long term^2^. Furthermore, frightened prey tend to forage less, leading to survival mechanisms like starvation, subsequently affecting birth and death rates^16–18^.

Whenever it is not always possible to experiment, recently, mathematical modeling has gained popularity as an effective tool for interpreting and studying infectious disease transmission. Mathematical modeling can offer insights relevant to such circumstances. This area which deals with ecological situations with an epidemic, is known as eco-epidemiology in mathematical biology. The effect of disease on the ecological system is a very important issue. Recently, mathematicians have been exploring ecological dynamics from the perspective of epidemiology. A SIRS system was first described by Kermack and Mckendrick^19^, in which the disease becomes transmissible through contact. First and foremost, Anderson and May^20^ looked at disease factors in prey-predator model. Most of the previous reviews discussed the dynamics of the predator–prey system characterized by disease in the prey population. These are cited, such as^20–28^. These studies were conducted with the aim of exterminating the infection in prey through adequate predation methods or other ecological effects. Thus, ecological models with infected predators are of the utmost importance when addressing the issue of predator conservation. Few cases have been reported where disease spread among predators, rather than among prey.

In 2002, Venturino^29^ investigated a predator–prey model involving disease within the predator population. Subsequently, in 2009, Haque and Pal^30,31^ examined a prey–predator model where an SIS parasitic infection exclusively affected the predator species. Their findings revealed a unique insight: despite the basic reproduction number for the prey species allowing invasion of the predator-only equilibrium, the presence of infection in the predator population could prevent its extinction. In 2014, Pal and Chakrabarti^31^ proposed a predator–prey model with diseases exclusively in predators. Their study demonstrated that, under specific predation levels, both prey and predator species could be rescued from extinction, as long as the disease did not spread within the predator population. Building upon this line of inquiry, Juneja et al.^32^ introduced an eco-epidemiological model with delays, assuming infection’s impact solely on predators. Their work factored in differential predation rates for infected and susceptible predators, along with distinct harvesting rates. Further developments emerged in 2019, when Huang et al.^33^ formulated a delayed SIS model, considering disease transmission only among predator species, while accounting for varied predator responses. Kumar et al.^34^ responded to a robust Allee effect in prey populations in 2020 by presenting an eco-epidemiological model. This model assumed disease propagation within predators exclusively and incorporated a ratio-dependent response function. Recently, in 2022, Dutta and Paul^35^ crafted a prey–predator model involving disease, fear factors, and additional food for efficient predators. Their model adopted a disease incidence rate inspired by the Beddington–DeAngelis type. Furthermore, Zhang et al.^36^ explored an eco-epidemic model with predator-borne diseases influenced by environmental noise. Despite these advancements, the field of mathematical eco-epidemiology has not extensively explored this topic. Consequently, mathematical epidemiology should dedicate more attention to the intricate dynamics of predator–prey interactions, especially when infections affect the predator population.

Defining the disease transmission term is a crucial aspect of epidemic modeling. In many epidemic models, disease spread is assumed to follow the law of mass action. If we denote the densities of susceptible and infectious populations at time *t* as *S*(*t*) and *I*(*t*) respectively, then, adhering to the mass action law (also known as bilinear law), the rate of new infections (or incidence rate) at any time *t* is characterized by $\beta (t) = \lambda g(I) S$, where $g(I) = I$. The coefficient $\lambda$ is termed the disease transmission coefficient. However, this mass action law presents certain unrealistic characteristics. Notably, the function *g*(*I*) becomes unbounded as *I* grows larger^37^. To address this limitation, Liu et al.^38^ proposed a nonlinear incidence rate and introduced a saturated nonlinear function for *g*(*I*), specifically $g(I) = \frac{\lambda I^p}{1 + bI^q}$, where *p*, *q* are positive constants, and *b* is a nonnegative constant. In this formulation, $\lambda I^p$ represents the force of infection, while $\frac{1}{1 + bI^q}$ accounts for inhibitory effects stemming from behavioral changes in infectious individuals as their numbers increase or due to the crowding impact caused by the presence of more infected individuals. Consequently, the incidence rate can be expressed as $\beta (t) = \frac{\lambda I^p S}{1 + bI^q}$. In their study of the cholera epidemic, Capasso and Serio^39^ considered an incidence rate of $\beta (t) = \frac{\lambda SI}{1 + bI}$ assuming $p=q=1$. It’s important to note that the crowding effect is negligible when *I* is small, and in such cases, the two infection rates become equivalent. Subsequently, various other researchers^38,40,41^ have adopted this incidence rate to analyze the dynamics of diverse epidemic models.

According to our knowledge, there are very few published studies on the effects of the treatment function on an eco-epidemiological model. However, none yet consider treatment for an eco-epidemic model where predators get infected. So, this gap needs to explore. Also, there are few eco-epidemic models where the effects of fear and refuge were considered but in those cases, infection was considered among prey species only^28,42^. But, none yet studied these effects by considering disease among predators. Here, is also a gap which need to explore. To our knowledge however, effects of fear and refuge in an infected predator–prey model with treatment has not yet been considered. Motivated by this discussion, in the present paper, we build a predator–prey model considering disease among the predator population only and due to fear of predator prey can take refuge. Additionally, we provide medical resources for treatment of infected individuals. The novelty of our work consists in the study of combined effects of fear and refuge in a prey–predator model where disease can spread among predators along with treatment. This is new model and none yet studied such type of model. This is our main contribution. The following ecological issues, we would like to explore in detail to describe the impact of our work:

1. What are the impacts of fear and refuge on system’s dynamics, specifically on infected species?
2. Whether treatment can control the disease or not?
3. Under which conditions disease can be eradicated from the system?
4. Is there any benefit of optimal control policy?
5. Are simultaneously disease-free and endemic steady states possible? If so, under which conditions do they exchange their stability?
6. When the ecosystem is in a coexistence state, what types of dynamics occur?

Though it is enough difficult to analyze or discuss all these questions because of it’s complexity, but, this investigation can result in a huge eco-epidemiological gain, and we will try to answer these questions by

1. obtaining system’s equilibria and complete stability analysis,
2. obtaining reproduction number analyzing possible bifurcation with respect to it,
3. obtaining global stability with respect to reproduction number,
4. obtaining any other possible bifurcations,
5. performing optimal control problem,
6. performing numerical simulation using Maple and MATLAB.

Our work is organized as follows: we developed an eco-epidemic model in “Model formulation” section by considering the fear effect and prey refuge. The treatment of infected populations was also considered. In “Preliminaries results” section, we have presented some preliminary results related to the mathematical analysis, including the positivity and boundedness of the solutions, the local stability of the system’s equilibria, and find the basic reproduction number. In “Extinction and persistence of the disease” section, we discussed the extinction and persistence of the disease such as Backward bifurcation and global stability of DFE (disease-free equilibrium), and the global stability of EE (Endemic equilibrium) points are analyzed. In “Bifurcation results, saddle-node and Hopf bifurcations of the system were discussed. The optimal control policy of the system is discussed in “Optimal control” section. In “Numerical analysis” section, all theoretical results are validated using numerical simulations. Finally, “Discussion and Conclusion” section concludes the paper with some discussion and conclusion.

## Model formulation

In order to formulate the mathematical model, assumptions are outlined below.

A1: In the considered predator–prey ecosystem, *X* and *Y* denote the total prey and predator populations.
A2: It has been assumed that predators are susceptible to some transmissible diseases. Disease in the predator population divides the whole predator population into two categories: (i) susceptible predators ($Y_s$) and (ii) infected predators ($Y_i$).
A3: We have assumed that when predators are absent, prey population grows logistically.
A4: Prey feels predation fear in the presence of predators. As a result, a linear portion of prey population takes refuge.
A5: We have considered that disease spreads among predator species only and the disease is not genetically inherited. The susceptible predator becomes infected under the attack of many viruses or parasites, respectively. Assume that the contact process follows a nonlinear interaction given by $\frac{\lambda Y_sY_i}{1+bY_i}$. There are compelling justifications for favoring a nonlinear incidence rate over the bilinear formulation ^43^. The mass action law lacks saturation and exhibits unbounded behavior as the infectious population, denoted as $Y_i$, increases significantly. Moreover, it neglects the inhibitory impact stemming from the behavioral changes in susceptible individuals and the crowding effect caused by the presence of infectious individuals^39^.
A6: Further, we assume that the infected predator population does not have the ability to reproduce as they are vulnerable to the disease. Also, we assume that infected predators can recover from the disease by their self-immunity.
A7: It is assumed that both predator populations predate the prey population following Holling type II functional form. But, the impact of disease ($e: \ 0<e<1$) acts on the predation of infected predator.
A8: Furthermore, we provide medical resources for treatment to the infected population. Taking into account the limitations of treatment capacity in different regions or communities, Wang^44^ introduced a staged treatment function that better represents real-world scenarios. This function states that the rate of treatment is proportional to the number of infectious individuals when the treatment capacity is not yet reached, and once the capacity is reached, the treatment rate saturates at its maximum level. This approach is more realistic compared to the conventional linear function. Additionally, it’s important to consider that treatment effectiveness can be compromised if infected individuals experience delays in receiving treatment. To capture the saturation effect in treatment, we adopt the treatment function $Trt(Y_i)=\frac{\gamma Y_i}{\beta +Y_i}$.

**Figure 1:**
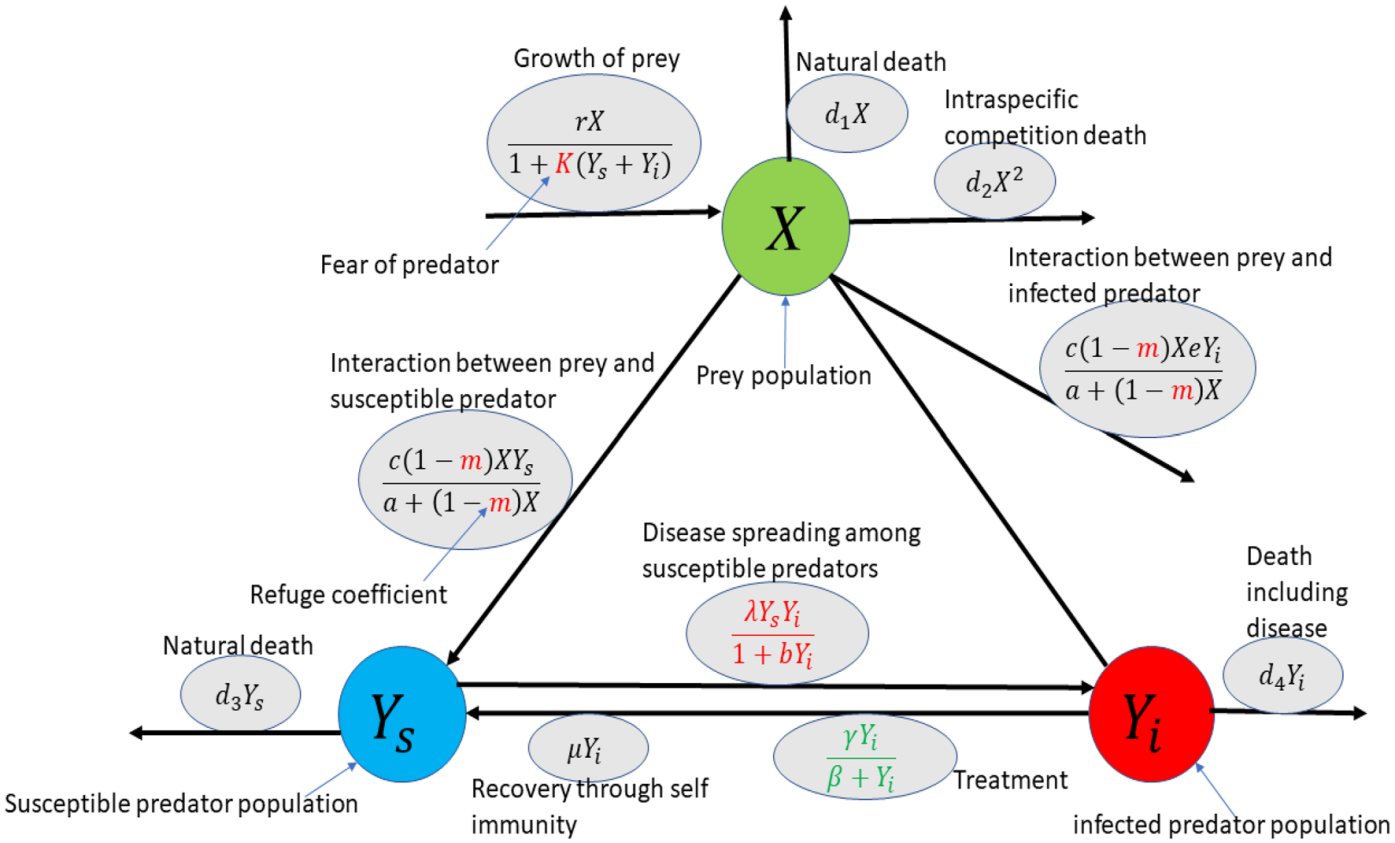
A complete schematic diagram describing growth, death and the dynamical interactions among prey (*X*), susceptible predator ($Y_s$) and infected predator ($Y_i$) in the context of disease, fear, refuge and treatment.

Keeping the above assumption in mind, we draw a schematic diagram, Fig. 1, which clearly shows all the interaction terms among the species. Based on this diagram, we formulate the following prey–predator model where predators get infection:

1 $$\begin{aligned} \frac{dX}{dt}= & {} \frac{rX}{1+K(Y_s+Y_i)}-d_1X-d_2X^2-\frac{c(1-m)X}{a+(1-m)X}(Y_s+eY_i),\nonumber \\ \frac{dY_s}{dt}= & {} \frac{c_1c(1-m)XY_s}{a+(1-m)X}-\frac{\lambda Y_sY_i}{1+bY_i}-d_3Y_s+\mu Y_i+\frac{\gamma Y_i}{\beta +Y_i},\nonumber \\ \frac{dY_i}{dt}= & {} \frac{\lambda Y_sY_i}{1+bY_i}-d_4 Y_i-\mu Y_i-\frac{\gamma Y_i}{\beta +Y_i}. \end{aligned}$$

Here, obviously $d_4\ge d_3$. Moreover, we assume $\lambda >d_4$, a biologically consistent phenomenon. The ecological significance of the model parameters is provided in Table 1. Moreover, to analyze system (1), the following initial conditions must be met:

2 $$\begin{aligned} {X(0)\ge 0, \ Y_s(0)\ge 0, \ Y_i(0)\ge 0.} \end{aligned}$$

**Table 1:** Eco-epidemiological description of the parameters involved in the model system (1), and their unit and value used for simulation.

Parameter	Biological meaning	Unit	Value
*r*	Growth rate of prey	$T^{-1}$	5.1
*K*	Fear of predators	$D^{-1}$	2.5
$d_1$	Natural death rate of prey	$T^{-1}$	0.25
$d_2$	Intraspecific death of prey	$D^{-1}T^{-1}$	0.15
*c*	Consumption rate of predators to prey	$T^{-1}$	0.25
*m*	Proportion of prey refuge	Unitless	0.2
*a*	Half saturation constant	*D*	0.7
*e*	Impact of disease on consumption rate	Unitless	0.41
$c_1$	Conversion efficiency of prey to susceptible predator	Unitless	0.25
$\lambda$	Disease prevalence rate	$D^{-1}T^{-1}$	0.5
*b*	Crowding effect of infected predators	$D^{-1}$	0.5
$d_3$	Natural death rate of predator	$T^{-1}$	0.011
$\mu$	Recovery rate of infected predator	$T^{-1}$	0.05
$\gamma$	Maximum medical resource supplied for treatment	$T^{-1}$	0.5
$\beta$	Stands for the saturation factor that measure the effect of	D	0.2
	the delay in treatment for the infected individuals		
$d_4$	Death rate of infected predator including natural and disease related death	$T^{-1}$	0.012

Here *T* represents the unit of time and *D* represents the unit of population density of a place.

## Preliminaries results

### Theorem 1

Solutions of the system (1) with initial condition (2) are non-negative for $t\ge 0$.

### Proof

Let $(X(t), Y_s(t), Y_i(t))$ be a solution of the system (1) with initial condition (2). We can derived from the first and third equations of system (1) that

$$\begin{aligned} X(t)= & {} X(0)e^{\int _{0}^{t}\left[ \frac{r}{1+K(Y_s+Y_i)}-d_1-d_2X-\frac{c(1-m)X}{a+(1-m)X}(Y_s+eY_i)\right] dz}\ge 0 \ \forall \ t{\ge 0}\\ Y_i(t)= & {} Y_i(0)e^{\int _{0}^{t}\left[ \frac{\lambda Y_s}{1+bY_i}-d_4-\mu -\frac{\gamma }{\beta +Y_i}\right] dz}{\ge 0} \ \forall \ t\ge 0. \end{aligned}$$

In order to demonstrate the positivity of $Y_s(t)$ on the interval $[0, \infty )$, we consider the case as follows.

We first assume that there exists a $t_1>0$ such that $Y_s(t_1)=0, \ {\dot{Y}}_s(t_1)<0$ and $Y_s(t)>0$ for $t\in [0, t_1)$. Therefore, from the second equation of system (1), we get

$$\begin{aligned} \frac{dY_s}{dt}\ge Y_s(0)e^{\int _{0}^{t}\left[ \frac{c_1c(1-m)X}{a+(1-m)X}-\frac{\lambda Y_i}{1+bY_i}-d_3\right] dz}{\ge 0}\ \forall \ t\ge 0. \end{aligned}$$

We get contradiction to the fact that ${\dot{Y}}_s(t_1)<0$. Thus, our assumption is not true. Hence, $Y_s(t){\ge 0}$ $\forall \ t\ge 0$. Therefore, solutions of the system (1) with non-negative initial state are always non-negative. $\square$

### Theorem 2

All solutions of system (1) initiating in ${\mathbb {R}}_+^3$ are uniformly bounded.

### Proof

Let $Z=c_1X+Y_s+Y_i$. Now, differentiating *Z* with respect to *t* along the solutions of system (1), we have

$$\begin{aligned} \frac{dZ}{dt}= & {} \frac{c_1rX}{1+K(Y_s+Y_i)}-c_1d_1X-c_1d_2X^2-\dfrac{c_1ce(1-m)XY_i}{a+(1-m)X}-d_3Y_s-d_4Y_i\\\le & {} c_1(rX-d_2X^2)-c_1d_1X-d_3Y_s-d_4Y_i\\\le & {} \frac{c_1r^2}{4d_2}-wZ, \ {[w=\min \{d_1, d_3, d_4\}]}. \end{aligned}$$

Therefore,

$$\begin{aligned} {0\le Z(X,Y_s,Y_i)\le \frac{c_1r^2}{4wd_2}(1-\exp ^{-wt})+Z(0)\exp ^{-wt}.} \end{aligned}$$

Taking limit $t\rightarrow 0$, we get $Z(X,Y_s,Y_i)\le \dfrac{c_1r^2}{4wd_2}$. Thus, every solution of system (1) initiating in ${\mathbb {R}}^3_+$ are contained in the region

$$\begin{aligned} { \Omega =\left\{ Z(X,Y_s,Y_i)\in {\mathbb {R}}^3_+: \ 0\le Z(X,Y_s,Y_i)\le \frac{c_1r^2}{4wd_2}+\varepsilon , \ \text {for any} \varepsilon >0\right\} .} \end{aligned}$$

$$\square$$

### System’s equilibria

**Figure 2:**
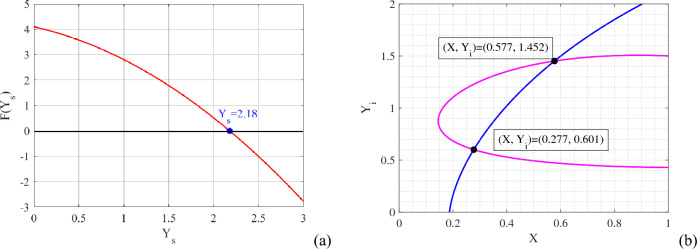
In figures, (**a**) red curve represents polynomial (3) and blue dot is the root of the polynomial; (**b**) magenta curve represents isocline (4) and blue curve represents isocline (5). From Table 1, the parameters’ values are selected.

The feasible equilibria of the system (1) are as follows:

1. The population-free equilibrium $E^0=(0,0,0)$, which always exists.
2. The predator-free equilibrium ${\widehat{E}}=\left( \dfrac{r-d_1}{d_2},0,0\right)$, which exists if $r>d_1$.
3. The disease-free equilibrium ${\overline{E}}=\left( {\overline{X}},{\overline{Y}}_s,0\right)$, where ${\overline{X}}=\dfrac{d_3a}{(c_1c-d_3)(1-m)}>0$ and ${\overline{Y}}_s$ is (are) the positive solution(s) of the following quadratic equation
  3 $$\begin{aligned} F(Y_s)\equiv AY_s^2+BY_s+C=0, \end{aligned}$$
  where $A=-c(1-m)K$, $B=-(d_1+d_2{\overline{X}})\{a+(1-m){\overline{X}}\}K-c(1-m)$ and $C=\{a+(1-m){\overline{X}}\}\{r-(d_1+d_2{\overline{X}})\}$. For the parameters value mentioned in Table 1, we can easily obtain the value of ${\overline{X}}=0.1868$ and for ${\overline{Y}}_s$, we plot the polynomial in Fig. 2a. From the figure, we find the value of ${\overline{Y}}_s=2.18$. Thus, for Table 1, we get a unique disease-free equilibrium ${\overline{E}}=(0.1868, 2.18, 0)$.
4. The endemic equilibrium ${\widetilde{E}}=({\widetilde{X}},{\widetilde{Y}}_s,{\widetilde{Y}}_i)$, where ${\widetilde{Y}}_s=\dfrac{1+b{\widetilde{Y}}_i}{\lambda }\left( d_4+\mu +\dfrac{\gamma }{\beta +{\widetilde{Y}}_i}\right) =G({\widetilde{Y}}_i)$ (say). ${\widetilde{X}}$ and ${\widetilde{Y}}_i$ are the positive solution(s) of the following isoclines
  4 $$\begin{aligned}{} & {} \dfrac{r}{1+K(G({\widetilde{Y}}_i)+{\widetilde{Y}}_i)}-d_1-d_2{\widetilde{X}}-\dfrac{c(1-m)}{a+(1-m){\widetilde{X}}}(G({\widetilde{Y}}_i)+e{\widetilde{Y}}_i)=0,\end{aligned}$$
  5 $$\begin{aligned}{} & {} \dfrac{c_1c(1-m){\widetilde{X}}G({\widetilde{Y}}_i)}{a+(1-m){\widetilde{X}}}-\dfrac{\lambda G({\widetilde{Y}}_i){\widetilde{Y}}_i}{1+b{\widetilde{Y}}_i}-d_3G({\widetilde{Y}}_i)+\mu {\widetilde{Y}}_i+\frac{\gamma {\widetilde{Y}}_i}{\beta +{\widetilde{Y}}_i}=0. \end{aligned}$$
  Mathematically, it is difficult to analyze the behavior of isoclines (4) and (5). Hence, we draw these isoclines in $X$ $-$ $Y_i$ plane to ensure the existence of endemic equilibrium (see Fig. 2b). It is depicted in the figure that there exist two intersecting points of the isoclines (4) and (5) for the parameters value mentioned in Table 1. These intersecting points give the endemic equilibrium points ${\widetilde{E}}=(0.277, 1.785, 0.601)$ and ${\widetilde{E}}=(0.577, 1.259, 1.452)$.

### Reproduction number

The reproduction number, often denoted as $R_0$, is a fundamental epidemiological concept employed to quantify the potential transmission of an infectious disease. It signifies the average count of new infections initiated by a single infected individual within a wholly susceptible population. To illustrate this concept with numerical values, following cases arise:

*Case-I:* $R_0 = 1$: This signifies that, on average, every infected individual will pass the virus to exactly one other person. In this scenario, the number of infections remains constant, indicating that the disease is endemic but not spreading rapidly.

*Case-II:* $R_0 < 1$: Each infected person, on average, will transmit the virus to less than one other person. Consequently, the number of infected individuals diminishes over time, leading to the eventual decline and extinction of the disease.

*Case-III:* $R_0 > 1$: In this situation, each infected individual, on average, will transmit the virus to more than one other person. As a result, the number of infected individuals increases, resulting in the rapid spread of the disease within the population. A higher $R_0$ value indicates a faster rate of disease propagation.

It is crucial to regulate the reproduction number to prevent exponential growth in infections during an outbreak (i.e., maintain it below 1 to bring the outbreak under control)^45,46^. The basic reproduction number ($R_0$) can be computed using the next-generation matrix formula. In this context, the following theorem is pertinent.

#### Theorem 3

System (1) has the following basic reproduction number

$$\begin{aligned} R_0=\dfrac{\lambda \beta {{\overline{Y}}}_s}{\beta (d_4+\mu )+\gamma }. \end{aligned}$$

#### Proof

There is only one infected population ($Y_i$) in system (1). In the system, third equation represents the dynamics of infected population. Infection term and remaining terms of that equation, respectively given by

$$\begin{aligned} F=\left( \dfrac{\lambda Y_s Y_i}{1+b Y_i}\right) _{1\times 1}, V=\left( d_4 Y_i+\mu Y_i +\dfrac{\gamma Y_i}{\beta +Y_i}\right) _{1\times 1}. \end{aligned}$$

Now, at the disease-free equilibrium ${{\overline{E}}}$, the next generation matrix calculated as

$$\begin{aligned} K=F V^{-1}=\left( \dfrac{\lambda \beta {{\overline{Y}}}_s}{\beta (d_4+\mu )+\gamma }\right) _{1\times 1}, \end{aligned}$$

where $F=\dfrac{\partial F}{\partial Y_i}$, $V=\dfrac{\partial V}{\partial Y_i}$. Therefore, basic reproduction number is given as $R_0=\dfrac{\lambda \beta {{\overline{Y}}}_s}{\beta (d_4+\mu )+\gamma }$. $\square$

### Local stability analysis

The Jacobian matrix of system (1) is given by $J=[a_{ij}]_{3\times 3}$, where

$$\begin{aligned} a_{11}= & {} \frac{r}{1+K(Y_s+Y_i)}-d_1-2d_2X-\frac{ac(1-m)(Y_s+eY_i)}{(a+(1-m)X)^2},\\ a_{12}= & {} -\frac{rKX}{(1+K(Y_s+Y_i))^2}-\frac{c(1-m)X}{a+(1-m)X}, a_{13}= -\frac{rKX}{(1+K(Y_s+Y_i))^2}-\frac{ec(1-m)X}{a+(1-m)X},\\ a_{21}= & {} \frac{ac_1c(1-m)Y_s}{(a+(1-m)X)^2},\ a_{22}=\frac{c_1c(1-m)X}{a+(1-m)X}-\frac{\lambda Y_i}{1+bY_i}-d_3,\\ a_{23}= & {} -\frac{\lambda Y_s}{(1+bY_i)^2}+\mu +\frac{\gamma \beta }{(\beta +Y_i)^2}, \ a_{31}=0, \ a_{32}=\frac{\lambda Y_i}{1+bY_i},\\ a_{33}= & {} \frac{\lambda Y_s}{(1+bY_i)^2}-(d_4+\mu )-\frac{\gamma \beta }{(\beta +Y_i)^2}. \end{aligned}$$

1. The eigenvalues of the Jacobian matrix $J(E^0)$ are obtained as $r-d_1$, -$d_3$ and $-\bigg (d_4+\mu +\frac{\gamma }{\beta }\bigg )$. $E^0$ is always stable if $r<d_1$ and unstable if $r>d_1$.
2. The eigenvalues of the Jacobian matrix $J({{\widehat{E}}})$ are obtained as $-(r-d_1)$, $\dfrac{c_1c(1-m)(r-d_1)}{d_2a+(1-m)(r-d_1)}-d_3$ and $-\bigg (d_4+\mu +\frac{\gamma }{\beta }\bigg )$. Thus, ${{\widehat{E}}}$ is stable if $r>d_1$ and $c_1c(1-m)(r-d_1)<d_3(d_2a+(1-m)(r-d_1))$, otherwise it is unstable.
3. Calculating at the Jacobian matrix at disease-free equilibrium ${{\overline{E}}}$, we obtain one eigenvalue as $\lambda \overline{Y}_s-(d_4+\mu +\dfrac{\gamma }{\beta })$, whereas other two are the roots of the following quadratic equation:
  $$\begin{aligned} \xi ^2+C_1\xi +C_0=0, \ \hbox {where} \ C_1=-(c_{11}+c_{22})\, \hbox {and} \ C_0=c_{11}c_{22}-c_{21}c_{12} \end{aligned}$$
  with
  $$\begin{aligned} c_{11}= & {} \frac{r}{1+K{{\overline{Y}}}_s}-d_1-2d_2\overline{X}-\frac{ac(1-m){{\overline{Y}}}_s}{(a+(1-m){{\overline{X}}})^2}, \ c_{22}=\frac{c_1c(1-m){{\overline{X}}}}{a+(1-m){{\overline{X}}}}-d_3,\\ c_{12}= & {} -{{\overline{X}}}\left( \dfrac{rK}{(1+K\overline{Y}_s)^2}+\frac{c(1-m)}{a+(1-m){{\overline{X}}}}\right) ,\ c_{21}=\frac{ac_1c(1-m){{\overline{Y}}}_s}{(a+(1-m){{\overline{X}}})^2}. \end{aligned}$$
  Therefore, ${{\overline{E}}}$ will be stable if $\lambda \overline{Y}_s<\bigg (d_4+\mu +\frac{\gamma }{\beta }\bigg )$, i.e., $R_0<1$ provided $C_1, \ C_0>0$. Otherwise it is unstable.
4. The Jacobian matrix at the equilibrium point ${{\widetilde{E}}}$ is given by $J_{{{\widetilde{E}}}}= [b_{ij}]_{3\times 3}$, where $b_{ij}=a_{ij}$ at ${{\widetilde{E}}}$. The characteristic equation of $J_{{{\widetilde{E}}}}$ is given by
  6 $$\begin{aligned} \xi ^3+B_2\xi ^2+B_1\xi +B_0=0, \end{aligned}$$
  where
  $$\begin{aligned} B_2= & {} -(b_{11}+b_{22}+b_{33}),\\ B_1= & {} b_{11}(b_{22}+b_{33})+b_{22}b_{33}-b_{12}b_{21}-b_{23}b_{32},\\ B_0= & {} b_{11}(b_{23}b_{32}-b_{22}b_{33})+b_{12}b_{21}b_{33}-b_{13}b_{21}b_{32}. \end{aligned}$$
  Using Routh–Hurwitz criteria, roots of (6) are negative or negative real part if $B_2>0$, $B_0>0$ and $B_1B_2-B_0>0$. Thus, for these conditions, ${{\widetilde{E}}}$ will be locally asymptotically stable, otherwise unstable.

## Extinction and persistence of the disease

### Backward bifurcation

#### Theorem 4

The system (1) undergoes backward bifurcation at $R_0=1$ whenever the sign of the coefficient $a'$ is positive where $a'$ is defined in (8).

#### Proof

We redefine the system (1) by changing variables as $X=x_1, Y_s=x_2$ and $Y_i=x_3$. Applying the vector notation $\textbf{x}=(x_1, x_2, x_3)^T$, the system (1) can be written as $\frac{d\textbf{x}}{dt}=\textbf{f}(\textbf{x})$ where $\textbf{f}=(f_1, f_2, f_3)^T$ as follows:

7 $$\begin{aligned} \frac{dx_1}{dt}= & {} f_1= \frac{rx_1}{1+K(x_2+x_3)}-d_1x_1-d_2x_1^2-\frac{c(1-m)x_1}{a+(1-m)x_1}(x_2+ex_3)\nonumber ,\\ \frac{dx_2}{dt}= & {} f_2= \frac{c_1c(1-m)x_1x_2}{a+(1-m)x_1}-\frac{\lambda x_2x_3}{1+bx_3}-d_3x_2+{\mu x_3}+\frac{\gamma x_3}{\beta +x_3},\nonumber \\ \frac{dx_3}{dt}= & {} f_3= \frac{\lambda x_2x_3}{1+bx_3}-d_4 x_3-\mu x_3-\frac{\gamma x_3}{\beta +x_3}. \end{aligned}$$

The disease free-equilibrium of the above system is given by ${{\overline{E}}}'({\overline{x}}_1, {\overline{x}}_2, 0 )$, where ${\overline{x}}_1=\frac{d_3a}{(c_1c-d_3)(1-m)}>0$ and ${\overline{x}}_2$ is (are) the positive solution(s) of the following equation $\dfrac{r}{1+K{\overline{x}}_2}-d_1-d_2{\overline{x}}_1-\dfrac{c(1-m){\overline{x}}_2}{a+(1-m){\overline{x}}_1}=0.$ Taking $\gamma$ as a bifurcation parameter we found $R_0(\gamma = \gamma ^*)=1$ which gives that the Jacobian of the transformed system (7) at the disease free equilibrium at $\gamma = \gamma ^*$ has a simple zero eigenvalue, while all other eigenvalues have negative real part.The Jacobian matrix of the above system at disease free equilibrium is given by

$$\begin{aligned} J({{\overline{E}}}') = \left( {\begin{array}{ccc} v_{11} &{} v_{12} &{} v_{13} \\ v_{21} &{} v_{22} &{} v_{23} \\ 0 &{} 0 &{} 0 \\ \end{array} } \right) , \end{aligned}$$

where

$$\begin{aligned} v_{11}= & {} \frac{r}{1+K{\overline{x}}_2}-d_1-2d_2{\overline{x}}_1-\frac{ac(1-m){\overline{x}}_2}{(a+(1-m){\overline{x}}_1)^2}, v_{12}= -\frac{rK{\overline{x}}_1}{(1+K{\overline{x}}_2)^2}-\frac{c(1-m){\overline{x}}_1}{a+(1-m){\overline{x}}_1},\\ v_{13}= & {} -\frac{rK{\overline{x}}_1}{(1+K{\overline{x}}_2)^2}-\frac{ec(1-m){\overline{x}}_1}{a+(1-m){\overline{x}}_1}, \ {v_{21}}= \frac{ac_1c(1-m){\overline{x}}_2}{(a+(1-m){\overline{x}}_1)^2}, \ {v_{22}}=\frac{c_1c(1-m){\overline{x}}_1}{a+(1-m){\overline{x}}_1}-d_3,\\ v_{23}= & {} -\lambda {\overline{x}}_2+\mu +\frac{\gamma }{\beta }. \end{aligned}$$

Now, we use the centre manifold theorem discussed by Castillo-Chavez and Song^47^ to study the dynamics of the system (7) near $\gamma = \gamma ^*$. The necessary computation for the theorem given below:

The right eigenvector of the Jacobian matrix $J({{\overline{E}}}')$ associated with zero eigenvalue at $\gamma =\gamma ^*$ is given by $W=(w_1, w_2, w_3)^T$ where, $w_1=\dfrac{v_{12}v_{23}-v_{13}v_{22}}{v_{11}v_{21}-v_{12}v_{21}}, w_2=\dfrac{v_{13}v_{21}-v_{11}v_{23}}{v_{11}v_{21}-v_{12}v_{21}}$ and $w_3=1$. It is also easy to see that the left eigenvector of the Jacobian matrix $J({{\overline{E}}}')$ associated with zero eigenvalue at $\gamma =\gamma ^*$ is given by $V=(v_1, v_2, v_3)$ where, $v_1=0, v_2=0$ and $v_3=1$ satisfying the condition $V.W=1$.

The bifurcation coefficient $a'$ and $b'$ at the disease free equilibrium and at $\gamma =\gamma ^*$ are given by

8 $$\begin{aligned} a'= & {} \sum _{k,i,j=1}^{3}v_k w_i w_j\frac{\partial ^2f_k}{\partial x_i\partial x_j}=v_1\sum _{i,j=1}^{3}w_i w_j\frac{\partial ^2f_1}{\partial x_i\partial x_j}+v_2\sum _{i,j=1}^{3}w_i w_j\frac{\partial ^2f_2}{\partial x_i\partial x_j}+v_3\sum _{i,j=1}^{3}w_i w_j\frac{\partial ^2f_3}{\partial x_i\partial x_j}\nonumber \\= & {} 2w_2\frac{\partial ^2f_3}{\partial x_2\partial x_3}+\frac{\partial ^2f_3}{\partial x_3^2}\nonumber \\= & {} 2\lambda (w_2-b)-d_4-\mu +\dfrac{\gamma ^*}{\beta }, \end{aligned}$$

$$\begin{aligned} b'= & {} \sum _{i,k=1}^{3} v_kw_i\frac{\partial ^2f_k}{\partial x_i\partial \gamma ^*}=v_1\left( w_1\frac{\partial ^2f_1}{\partial x_1\partial \gamma ^*}+w_2\frac{\partial ^2f_1}{\partial x_2\partial \gamma ^*}+w_3\frac{\partial ^2f_1}{\partial x_3\partial \gamma ^*}\right) +v_2\left( w_1\frac{\partial ^2f_2}{\partial x_1\partial \gamma ^*}+w_2\frac{\partial ^2f_2}{\partial x_2\partial \gamma ^*}+w_3\frac{\partial ^2f_2}{\partial x_3\partial \gamma ^*}\right) \\&\quad +&v_3\left( w_1\frac{\partial ^2f_3}{\partial x_1\partial \gamma ^*}+w_2\frac{\partial ^2f_3}{\partial x_2\partial \gamma ^*}+w_3\frac{\partial ^2f_3}{\partial x_3\partial \gamma ^*}\right) \\= & {} \frac{\partial ^2f_3}{\partial x_3\partial \gamma ^*}=-\dfrac{1}{\beta }\ne 0. \end{aligned}$$

Hence, following Theorem 4 in^48^, the system (1) undergoes backward bifurcation at $R_0=1$ whenever $a'>0$. $\square$

#### Theorem 5

For $R_0<1$, the disease-free equilibrium ${{\overline{E}}}$ is globally asymptotically stable in the interior of the positive quadrant of $X-Y_s$ plane of the system (1) if $\overline{Y_s}<\dfrac{a^2d_2}{c(1-m)^2}$.

#### Proof

The global asymptotically stable of the boundary equilibrium point ${{\overline{E}}}({\overline{X}}, \overline{Y_s}, 0)$ is shown with the help of Lyapunov function. Let us consider a Lyapunov function ${\overline{V}}$ as,

$$\begin{aligned} {\overline{V}}=\left( X-{\overline{X}}-{{\overline{X}}}\ln \frac{X}{\overline{X}}\right) +\sigma \left( Y_s-{{\overline{Y}}}_s-\overline{Y}_s\ln \frac{Y_s}{{{\overline{Y}}}_s}\right) , \end{aligned}$$

where $\sigma$ is a positive constant which will be chosen later. Taking trajectory derivative of this scalar valued function along the solutions of the disease-free subsystem of the system (1), we get

$$\begin{aligned} \frac{d{\overline{V}}}{dt}= & {} \left( \dfrac{X-\overline{X}}{X}\right) \dfrac{dX}{dt}+\sigma \left( \dfrac{Y_s-\overline{Y_s}}{Y_s}\right) \dfrac{dY_s}{dt}\\\le & {} [-d_2+\frac{c(1-m)^2}{a^2}\overline{Y_s}](X-{{\overline{X}}})^2\\ ~~~~~~~~~~{} & {} +\left[ -\frac{c(1-m)}{a+(1-m)X}+\dfrac{\sigma ac_1c(1-m)}{(a+(1-m)X)(a+(1-m)\overline{X})}\right] (X-{\overline{X}})(Y_s-\overline{Y_s}). \end{aligned}$$

Now, we choose the positive constant $\sigma =\dfrac{a+(1-m){\overline{X}}}{ac_1}$, then above equation can be written as,

$$\begin{aligned} \frac{d{\overline{V}}}{dt}\le & {} [-d_2+\frac{c(1-m)^2}{a^2}\overline{Y_s}](X-{{\overline{X}}})^2< 0~~~~ (\text {by the condition given in the theorem}). \end{aligned}$$

Therefore, $\dfrac{d{\overline{V}}}{dt}$ is negative definite function along all the trajectories in $X-Y_s$ plane. Let *M* be the largest invariant subset of the set $\Omega =\{(X(t), Y_s(t),Y_i(t))\Big \vert \frac{d{\overline{V}}}{dt}=0\}$. We now claim that $M=\{{\overline{E}}\}$. In fact when $R_0<1$, we see that $\Omega =\{(X(t), Y_s(t),Y_i(t))\Big \vert X(t)={\overline{X}}, Y_s(t)=\overline{Y_s}, Y_i(t)=0\}$, which yields $M=\{{\overline{E}}\}$. Note that *M* is invariant. By applying LaSalle’s invariance principle for delay differential equations^49^, we establish the global asymptotic stability of the disease-free equilibrium ${\overline{E}}$ under the specific conditions delineated in the theorem. $\square$

#### Theorem 6

For $R_0>1$, the endemic equilibrium ${{\widetilde{E}}}$ is globally asymptotically stable if following conditions are satisfied

$$\begin{aligned}&V_{11}>0, V_{11}V_{22}-V^2_{11}>0, \\&V_{11}(V_{22}V_{33}-V^2_{23})-V_{12}(V_{12}V_{33}-V_{13}V_{23})+ V_{13}(V_{12}V_{23}-V_{13}V_{22})>0. \end{aligned}$$

#### Proof

In order to prove this theorem, we consider a Lyapunov function *V* that is positive definite

$$\begin{aligned} V=\left( X-{{\widetilde{X}}}-{{\widetilde{X}}}\ln \frac{X}{\widetilde{X}}\right) +\left( Y_s-{{\widetilde{Y}}}_s-\widetilde{Y}_s\ln \frac{Y_s}{{{\widetilde{Y}}}_s}\right) +\left( Y_i-\widetilde{Y}_i-{{\widetilde{Y}}}_i\ln \frac{Y_i}{{{\widetilde{Y}}}_i}\right) . \end{aligned}$$

Now calculating the time derivative of *V* along the solution of the system (1), we get

$$\begin{aligned} \frac{dV}{dt}={} & {} -\{V_{11}(X-{{\widetilde{X}}})^2+V_{22}(Y_s-\widetilde{Y}_s)^2+V_{33}(Y_i-{{\widetilde{Y}}}_i)^2+2V_{12}(X-\widetilde{X})(Y_s-{{\widetilde{Y}}}_s)\\{} & {} + 2V_{13}(X-{{\widetilde{X}}})(Y_i-\widetilde{Y}_i)+2V_{23}(Y_s-{{\widetilde{Y}}}_s)(Y_i-{{\widetilde{Y}}}_i)\}\\ ={} & {} -W^TV^*W, \end{aligned}$$

where $W=((X-{{\widetilde{X}}})\ \ (Y_s-{{\widetilde{Y}}}_s)\ \ (Y_i-{{\widetilde{Y}}}_i))^T$ and $V^*$ is a symmetric matrix given by

$$\begin{aligned} V^* = \left( {\begin{array}{ccc} V_{11} &{} V_{12} &{} V_{13} \\ V_{12} &{} V_{22} &{} V_{23} \\ V_{13} &{} V_{23} &{} V_{33} \\ \end{array} } \right) \end{aligned}$$

with

$$\begin{aligned} V_{11}& = d_2-\frac{(1-m)(Y_s+Y_i)}{(a+(1-m)X)(a+(1-m){{\widetilde{X}}})}, V_{22}=\frac{d_3}{Y_s}+\frac{\lambda \widetilde{Y}_i}{Y_s(1+b{{\widetilde{Y}}}_i)}-\frac{c_1c(1-m)\widetilde{X}}{Y_s(a+(1-m){{\widetilde{X}}})},\\ V_{33}& = \frac{\lambda bY_s}{(1+bY_i)(1+b\widetilde{Y}_i)}-\frac{\gamma }{(\beta +Y_i)(\beta +{{\widetilde{Y}}}_i)},\\ V_{12}& = -\frac{c_1c(1-m)}{(a+(1-m)X)(a+(1-m)\widetilde{X})}+\frac{c(1-m)}{a+(1-m)\widetilde{X}}+\frac{kr}{(1+k(Y_s+Y_i))(1+k({{\widetilde{Y}}}_s+{{\widetilde{Y}}}_i))},\\ V_{13}& = \frac{kr}{(1+k(Y_s+Y_i))(1+k({{\widetilde{Y}}}_s+\widetilde{Y}_i))}+\frac{ce(1-m)}{a+(1-m){{\widetilde{X}}}},\\ V_{23}& = \frac{\lambda }{(1+bY_i)(1+b\widetilde{Y}_i)}-\frac{\mu }{Y_s}-\frac{\gamma \beta }{Y_s(\beta +Y_i)(\beta +\widetilde{Y}_i)}-\frac{\lambda }{1+b{{\widetilde{Y}}}_i}. \end{aligned}$$

Note that under given conditions specified in the theorem, $V^*$ is positive definite matrix, then $\dfrac{dV}{dt}$ will be negative definite. Hence, for $R_0>1$, ${{\widetilde{E}}}$ is globally asymptotically stable according to the Lyapunov stability theorem. $\square$

*Remark* From a biological perspective, the global stability of disease-free equilibrium points and endemic equilibrium points are of the utmost importance. As a result of the analysis, we obtain conditions under which diseases may or may not survive in the system. Moreover, in these situations, regardless of population size, either disease will persist or not.

## Bifurcation results

### Saddle-node bifurcation analysis

System (1) have at most two endemic equilibrium points, depending on *m*. Assume that the two endemic equilibrium points coincide at $m=m^{[SN]}$. On one side of $m=m^{[SN]}$, there are two endemic equilibrium points, while on the other there are no endemic equilibrium points. Consequently, the saddle-node bifurcation may occur around the endemic equilibrium point when the system parameter *m* crosses its bifurcation threshold. In the following theorem, we shall establish the conditions for the occurrence of saddle-node bifurcation concerning *m* as the bifurcation parameter.

#### Theorem 7

System (1) undergoes saddle-node bifurcation at the coincident endemic equilibrium ${{\widetilde{E}}}$ when it goes through the critical value $m=m^{[SN]}$ and satisfy following conditions

9 $$\begin{aligned} c_1h_{11}{\widetilde{Y}}_i+h_{21}({\widetilde{Y}}_s+e{\widetilde{Y}}_i)\ne 0 \ and \ E_1W_1+E_2W_2+E_3W_3 \ne 0. \end{aligned}$$

#### Proof

We must use Sotomayor’s theorem in order to establish dynamics of the system in the neighbourhood of the equilibrium point $\widetilde{E}$ since the eigenvalue of $J({{\widetilde{E}}})$ is zero at $m=m^{[SN]}$. The following function is considered for this purpose:

$$\begin{aligned} F(Z,m)=\left[ F_1(Z,m), \ F_2(Z,m), \ F_3(Z,m)\right] ^T, \text {where} \ Z=[X \ Y_s \ Y_i]^T, \end{aligned}$$

and $F_1$, $F_2$ and $F_3$ represent right hand sides of first, second and third equations of system (1), respectively.

Then, we have

$$\begin{aligned} F_m(Z={{\widetilde{E}}}, m=m^{[SN]})=\left[ \begin{array}{lll} \dfrac{acX({\widetilde{Y}}_s + e{\widetilde{Y}}_i)}{\{a+(1-m^{[SN]}){\widetilde{X}}\}^2}&-\dfrac{ac_1c{\widetilde{X}}{\widetilde{Y}}_s}{\{a+(1-m^{[SN]}){\widetilde{X}}\}^2}&0 \end{array}\right] ^T \end{aligned}$$

and $DF(Z,m)=J=[a_{ij}]_{3\times 3}$. Therefore, $DF(Z={{\widetilde{E}}}, m=m^{[SN]})=[h_{ij}]_{3\times 3}$ (say), where $h_{ij}=b_{ij}$ evaluated for $m=m^{[SN]}$. Let $V=[V_1 \ V_2 \ V_3]^T$ and $W=[W_1 \ W_2 \ W_3]^T$ are the eigenvectors corresponding to the eigenvalue zero of the matrix $DF(Z={{\widetilde{E}}}, m=m^{[SN]})$ and its transpose, then we have

$$\begin{aligned} V_1=\frac{h_{12}h_{33}-h_{13}h_{32}}{h_{32}h_{11}}, \ V_2=\frac{h_{33}}{h_{32}}, V_3=1=W_1, \ W_2=-\frac{h_{11}}{h_{21}}, \ W_3=\frac{h_{12}h_{21}-h_{22}h_{11}}{h_{32}h_{21}}. \end{aligned}$$

Now,

$$\begin{aligned}&D^2F(Z,m)(V,V)=[D_1 \ D_2 \ D_3]^T\\ {}&=\left( \begin{array}{ll}\dfrac{\partial ^2F_1}{\partial X^2}V_1^2+\dfrac{\partial ^2F_1}{\partial Ys^2}V_2^2+\dfrac{\partial ^2F_1}{\partial Yi^2}V_3^2+2\dfrac{\partial ^2F_1}{\partial X\partial Ys}V_1V_2+2\dfrac{\partial ^2F_1}{\partial X\partial Yi}V_1V_3+2\dfrac{\partial ^2F_1}{\partial Ys\partial Yi}V_2V_3\\ \dfrac{\partial ^2F_2}{\partial X^2}V_1^2+\dfrac{\partial ^2F_2}{\partial Ys^2}V_2^2+\dfrac{\partial ^2F_2}{\partial Yi^2}V_3^2++2\dfrac{\partial ^2F_2}{\partial X\partial Ys}V_1V_2+2\dfrac{\partial ^2F_2}{\partial X\partial Yi}V_1V_3+2\dfrac{\partial ^2F_2}{\partial Ys\partial Yi}V_2V_3\\ \dfrac{\partial ^2F_3}{\partial X^2}V_1^2+\dfrac{\partial ^2F_3}{\partial Ys^2}V_2^2+\dfrac{\partial ^2F_3}{\partial Yi^2}V_3^2++2\dfrac{\partial ^2F_3}{\partial X\partial Ys}V_1V_2+2\dfrac{\partial ^2F_3}{\partial X\partial Yi}V_1V_3+2\dfrac{\partial ^2F_3}{\partial Ys\partial Yi}V_2V_3\end{array} \right) . \end{aligned}$$

Thus,

$$\begin{aligned} D^2F\left( Z={{\widetilde{E}}}, m=m^{[SN]}\right) (V,V)=[E_1 \ E_2 \ E_3]^T, \text {where} \ E_i=D_i\left( Z={{\widetilde{E}}}, m=m^{[SN]}\right) , \ i=1,2,3. \end{aligned}$$

Now,

$$\begin{aligned} W^TF_m\left( Z={{\widetilde{E}}}, m=m^{[SN]}\right)= & {} \frac{ac{\widetilde{X}}}{\{a+(1-m^{[SN]}){\widetilde{X}}\}^2h_{21}}\{c_1h_{11}{\widetilde{Y}}_i +h_{21}({\widetilde{Y}}_s+e{\widetilde{Y}}_i)\}\\ W^TD^2F\left( Z={{\widetilde{E}}}, m=m^{[SN]}\right) (V,V)= & {} E_1W_1+E_2W_2+E_3W_3. \end{aligned}$$

In other words, if the conditions stated in the theorem are met, system (1) experiences saddle-node bifurcation around the coincident endemic equilibrium point ${{\widetilde{E}}}$. $\square$

To justify the above result numerically, we have considered the values of all the parameters from Table 1 except $K=2.8$ and *m*. At $m=m^{[SN]}=0.9128162$, the system (1) has a coincident endemic equilibrium ${{\widetilde{E}}}(2.263014968,0.4605346438,2.015085770)$. The corresponding characteristic matrix

$$J( {{\widetilde{E}}})|_{m=m^{[SN]}}= \left( \begin{matrix} -\,0.3278528942 &{} -\,0.5691363783&{}-\,0.5366913706\\ 0.009538321630 &{} -\,0.1846246896&{}-\,0.3861678473\\ 0&{}0.1873725742&{}0.3741678473 \end{matrix} \right)$$ has a simple zero eigenvalue. Eigenvectors *V* and *W* corresponding to the zero eigenvalue of the matrices $J(\widetilde{E})$and $J({{\widetilde{E}}})^T$ are obtained as $V=\left( 1.828943964, -1.996696027,1\right) ^T$ and $W=\left( 1,34.32119970,36.84875085\right) ^T$. Moreover, $F_m\left( {{\widetilde{E}}}, m^{[SN]}\right) =\left( 1.084038494, -\,0.2477909176, 0\right) ^T$. This yields,

1. $$W^TF_c\left( {{\widetilde{E}}}, m^{[SN]}\right) =-\,7.420443073\ne 0$$,
2. $$W^TD^2F\left( {{\widetilde{E}}}, m^{[SN]}\right) (V, V)=-\,4.027014714\ne 0$$.

Therefore, according to Sotomayor’s theorem^50^, system (1) approaches a saddle-node bifurcation when the parameter *m* crosses the critical value $m=m^{[SN]}=0.9128162$.

### Hopf bifurcation

#### Theorem 8

System (1) possesses Hopf bifurcation if $(B_1-3\omega ^2)\bigg (\frac{dB_0}{dm}-\omega ^2\frac{dB_2}{dm}\bigg )+2B_2\omega ^2\frac{dB_1}{dm}\ne 0$ when *m* crosses the critical value $m=m^{[HB]}$.

#### Proof

Hopf bifurcation in our model arises when one of the eigenvalues of the variation matrix $J({{\widetilde{E}}})$ has negative real part with $Re(\frac{d\xi }{dm})_{m=m^{[HB]}}\ne 0$ and other two eigenvalues are purely imaginary. The characteristic Eq. (6) must satisfy the conditions $B_2B_1-B_0=0$ for purely imaginary eigenvalues. A purely imaginary eigenvalue is assumed to be $i\omega$ for $m^{[HB]}$.

Differentiating the characteristic Eq. (6) with respect to *m*, we obtain

$$\begin{aligned} (3\xi ^2+2B_2\xi +B_1){\frac{d\xi }{dm}}+{\xi ^2\frac{dB_2}{dm}}+{\xi \frac{dB_1}{dm}}+{\frac{dB_0}{dm}}=0 \\ \frac{d\xi }{dm}=-\frac{\xi ^2\frac{dB_2}{dm}+\xi \frac{dB_1}{dm}+\frac{dB_0}{dm}}{3\xi ^2+2B_2\xi +B_1 }. \end{aligned}$$

Now, putting $\xi =i\omega$ and comparing with real part, we have

$$\begin{aligned} \left( Re\left( \frac{d\xi }{dm}\right) \right) _{\xi =i\omega }=-\frac{(B_1-3\omega ^2) \left( \frac{dB_0}{dm}-\omega ^2\frac{dB_2}{dm}\right) +2B_2\omega ^2\frac{dB_1}{dm}}{(B_1-3\omega ^2)^2+(2B_2\omega )^2}. \end{aligned}$$

Thus, system (1) experiences a Hopf bifurcation with respect to the parameter *m* if condition

$$(B_1-3\omega ^2)\left( \frac{dB_0}{dm}-\omega ^2\frac{dB_2}{dm}\right) +2B_2\omega ^2\frac{dB_1}{dm}\ne 0$$

satisfies at $$m=m^{[HB]}$$. $$\square$$

As it is very difficult to follow the Hopf bifurcation result analytically, so, we verify it numerically. We have taken the values of all the parameters from Table 1 except *m*. Calculating $B_1B_2-B_0=0$ for *m*, we find the critical value of *m* as $m^{[HB]}=0.23058$. Then at this critical value Eq. (6) becomes as follows:

10 $$\begin{aligned} \xi ^3+0.2802\xi ^2+0.0045\xi +0.0013=0. \end{aligned}$$

Solving this equation, we get one of the eigenvalues as $i\omega$ (where $\omega =0.0673$), which is purely imaginary.

Now, we calculate the expression $Re\left( \frac{d\xi }{dm}\right)$ at $\xi =i\omega$ and get $7.36 \ (\ne 0)$. Thus, the transversality condition verified at $m=m^{[HB]}$. Therefore, this numerical example confirms the existence of Hopf bifurcation of system (1) at $m=m^{[HB]}$.

## Optimal control

Controlling the predator–prey system is possible if humans are able to reach a certain limit. To prevent infection on predator, control is applied in this model. The purpose of the optimal control is to optimize the size of susceptible predator and minimize the cost of treatment of infected individuals. A problem of optimal control is considered in which the objective function should be minimized as follows:

$$\begin{aligned} J(\gamma )=\int _{0}^{T}(AY_i(t)+B\gamma ^2(t))dt, \end{aligned}$$

where *A* and *B* are respectively weighted constants per capita less due to presence of infected population and treatment of infected individuals. Our problem is to find optimal control function $(\gamma ^*(t))$ such that

11 $$\begin{aligned} J(\gamma ^*(t))=\min \{J(\gamma ),\ \gamma \in U\}, \ \text {where} \ \ U=\{\gamma (t)| \ 0\le \gamma (t)\le 1,\ t\in [0, T]\}. \end{aligned}$$

The Lagrangian of the given problem (1) is to defined as $L=AY_i+B\gamma ^2$. Now, we form the Hamiltonian *H* for the problem is given by

$$\begin{aligned} H(X,Y_s,Y_i,\gamma ,\lambda _1,\lambda _2,\lambda _3){} & {} =AY_i+B\gamma ^2\\{} & {} \quad +\lambda _1(t)\left\{ \dfrac{rX}{1+K(Y_s+Y_i)}-d_1X-d_2X^2-\dfrac{c(1-m)X}{a+(1-m)X}(Y_s+eY_i)\right\} \\{} & {} \quad +\lambda _2(t)\left\{ \dfrac{c_1c(1-m)XY_s}{a+(1-m)X}-\dfrac{\lambda Y_sY_i}{1+bY_i}-d_3Y_s+\mu Y_i+\frac{\gamma Y_i}{\beta +Y_i}\right\} \\{} & {} \quad +\lambda _3(t)\left\{ \dfrac{\lambda Y_sY_i}{1+bY_i}-d_4 Y_i-\mu Y_i-\frac{\gamma Y_i}{\beta +Y_i}\right\} . \end{aligned}$$

Using Pontryagin’s Maximum Principle, the adjoint equations are given by

$$\begin{aligned} \dfrac{d\lambda _1}{dt}=-\dfrac{\partial H}{\partial X}, \ \dfrac{d\lambda _2}{dt}=-\dfrac{\partial H}{\partial Y_s} \ \text {and} \ {\dfrac{d\lambda _3}{dt}=-\dfrac{\partial H}{\partial Y_i}} \end{aligned}$$

with the transversality conditions $\lambda _i(T)=0,\ \ \ i=1,2,3$.

Thus, we have

12 $$\begin{aligned} \dfrac{d\lambda _1}{dt}{} & {} =\lambda _1\left( -\dfrac{r}{1+K(Y_s+Y_i)}-d_1-2d_2X-\frac{ac(1-m)(Y_s+eY_i)}{(a+(1-m)X)^2}\right) \nonumber \\{} & {} \quad -\dfrac{\lambda _2c_1ca(1-m)Y_s}{(a+(1-m)X)^2}\nonumber ,\\ \frac{d\lambda _2}{dt}{} & {} =\dfrac{\lambda _1rKX}{(1+K(Y_s+Y_i))^2}+\dfrac{c(1-m)X}{a+(1-m)X}\{\lambda _1-c_1\lambda _2\}+\lambda _2\left( \dfrac{\lambda Y_i}{1+bY_i}+d_3\right) \\{} & {} \quad -\dfrac{\lambda Y_i}{1+bY_i}\nonumber ,\\ \frac{d\lambda _3}{dt}{} & {} =-A+\lambda _1\left( \dfrac{ec(1-m)X}{a+(1-m)X}-\dfrac{rKX}{(1+K(Y_s+Y_i))^2}\right) +\dfrac{(\lambda _3-\lambda _2)\beta \gamma }{(\beta +Y_i)^2}\nonumber \\{} & {} \quad +\dfrac{(\lambda _2-\lambda _3)\lambda Y_s}{(1+bY_i)^2}+(\lambda _3-\lambda _2)\mu +\lambda _3d_4\nonumber \end{aligned}$$

with the transversality conditions

13 $$\begin{aligned} \lambda _1(T)=0,~~~\lambda _2(T)=0,~~~\lambda _3(T)=0. \end{aligned}$$

Now, using the optimality condition $\dfrac{\partial H}{\partial \gamma }=0$, we get $\gamma =\dfrac{(\lambda _3-\lambda _2)Y_i}{2B(\beta +Y_i)}$.

The value of $\gamma$ is in the interval [0, 1] so that some possibilities are obtained below

14 $$\begin{aligned} \gamma ^*={\left\{ \begin{array}{ll} 0, ~~~&{} \text{ if } ~~~~~ \frac{(\lambda _3-\lambda _2)Y_i}{2B(\beta +Y_i)}\le 0 \\ \frac{(\lambda _3-\lambda _2)Y_i}{2B(\beta +Y_i)}, ~~~&{} \text{ if } ~~~~~ 0<\frac{(\lambda _3-\lambda _2)Y_i}{2B(\beta +Y_i)}< 1 \\ 1, ~~~&{} \text{ if }~~~~~ \frac{(\lambda _3-\lambda _2)Y_i}{2B(\beta +Y_i)}\ge 1. \end{array}\right. } \end{aligned}$$

Thus, the following optimal control variable is obtained

$$\begin{aligned} \gamma ^*=\min \left( \max \left( 0,~ \frac{(\lambda ^*_3-\lambda ^*_2)Y^*_i}{2B(\beta +Y^*_i)}\right) ,~ 1\right) . \end{aligned}$$

Here, $X^*, Y^*_s, Y^*_i$ are respectively optimum value of $X, Y_s, Y_i$ and $(\lambda ^*_1, \lambda ^*_2, \lambda ^*_3)$ is solution of the system (12) with the condition (13). In the following theorem, we summarize the details.

### Theorem 9

The optimal control $\gamma ^*$ which minimize the objective function *J* over the region *U* shown in (11) are given by $\gamma ^*=\min \left( \max \left( 0,~ \dfrac{(\lambda ^*_3-\lambda ^*_2)Y^*_i}{2B(\beta +Y^*_i)}\right) ,~ 1\right)$.

### Numerical results

To explore the insight dynamics of the system (1) and verify analytical results, we perform extensive numerical simulations in this section. Simulations are performed in MATLAB and numerical continuation software MAPLE. In our numerical simulations, we have selected a collection of biologically plausible hypothetical parameter values, which are outlined in Table 1. Although these values are hypothetical in nature, they are drawn from existing literature sources^27,28,41,42,51,52^. Notably, these values closely resemble those found in these existing works.

**Figure 3:**
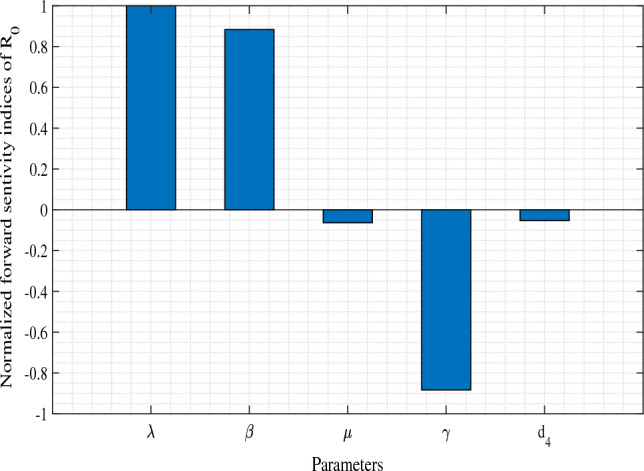
Normalized forward sensitivity indices of $R_0$. Parameters values are: $\lambda =0.5$, $\beta =0.2$, $\mu =0.18$, $\gamma =0.5$, and $d_4=0.15$.

In order to determine the influence of parameters on $R_0$, we compute normalized forward sensitivity indices for $R_0$^53^. An index of normalized forward sensitivity measures how much the variable has changed based on changes in the parameter. In Fig. 3, a plot is displayed showing the normalized forward sensitivity indices for each parameter in the expression of $R_0$. This figure illustrates that the value of $R_0$ increases as the parameters $\lambda$ and $\beta$ have positive indices. On the other hand, parameters $\mu$, $\gamma$ and $d_4$ possess negative indices with $R_0$. Figure 3, reveals that the parameters $\lambda$, $\beta$ and $\gamma$ are most sensitive to $R_0$. It is important to note that we prefer low values of $R_0$ as they enhance the possibility of eradicating the disease. The increment of parameters $\lambda$ and $\beta$ should therefore be prevented at all costs. On the other hand, the increment of $\mu$, $\gamma$, and $d_4$ should be encouraged. Therefore, in order to eradicate disease, policymakers should focus on preventing an increase in the parameters with positive indices, while stimulating the parameters with negative indices.

**Figure 4:**
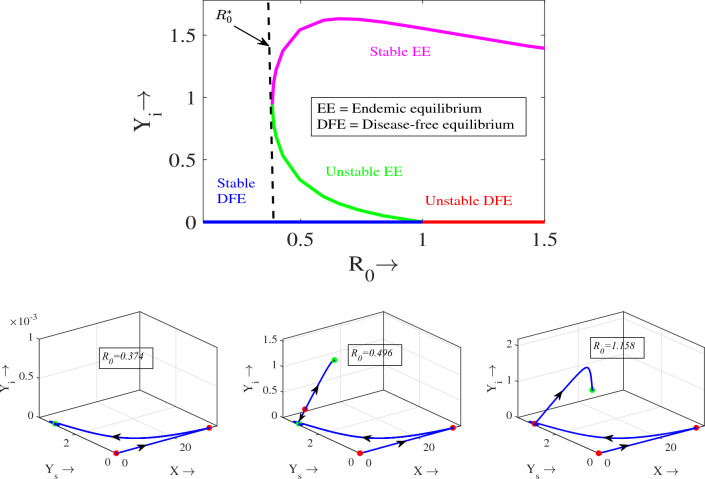
First row: Backward bifurcation with respect to $R_0$. Second row: Phase portraits for different values of $R_0$. Parameters have the same values as in Table 1 except $m=0.7$.

**Figure 5:**
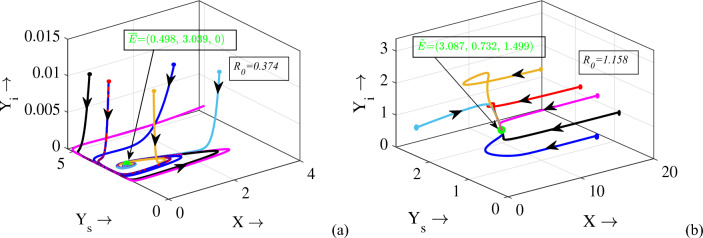
Solution trajectories of system (1) in $X-Y_s-Y_i$ space for different initial values. Parameters are at the same values as in Table 1 except $m=0.7$. In the figures, green dots represent the equilibrium points. Circles in each trajectory describe the initial start positions of the trajectories.

The justification for the Backward bifurcation can be visually grasped through a one-parameter bifurcation diagram utilizing $R_0$ as a basis. As evident from Theorem 4, the exploration of backward bifurcations is contingent upon the parameter $\gamma$. This relationship is represented graphically in terms of $R_0$ in Fig. 4. The diagram clearly indicates that the disease-free equilibrium point is stable if $R_0 < 1$ and unstable if $R_0 > 1$. Conversely, for $R_0>1$, only one endemic equilibrium (${\widetilde{E}}$) exists and is stable. Furthermore, there exists a range of $R_0^*< R_0 < 1$ (with $R_0^* < 1$), where a significant phenomenon arises. In this interval, the system exhibits two interior (endemic) equilibrium points. Among these points, the one characterized by a higher infected density is stable, while the other is unstable. This scenario leads to a state of bistability, where both the DFE and the EE stable simultaneously. The second row of Fig. 4 visually illustrates these scenarios through phase portraits. In the first figure (where $R_0<R_0^*$), all trajectories converge to the disease-free state; in the second figure (where $R_0\in [R_0^*,1]$), the trajectories’ convergence depends on initial population size, with some reaching the disease-free state and others the endemic state; in the last figure (where $R_0>1$), all trajectories converge to the endemic state. Further, insights can be gleaned by considering the specific ranges of $R_0$. For $R_0<R_0^*$, only the DFE exists and is globally asymptotically stable, as depicted in Fig. 5a. Conversely, for $R_0>1$, one endemic equilibrium (${\widetilde{E}}$) exists and is globally asymptotically stable, as evident from Fig. 5b. The Backward bifurcation thus plays a pivotal role in comprehending the dynamics of disease within an ecosystem.

**Figure 6:**
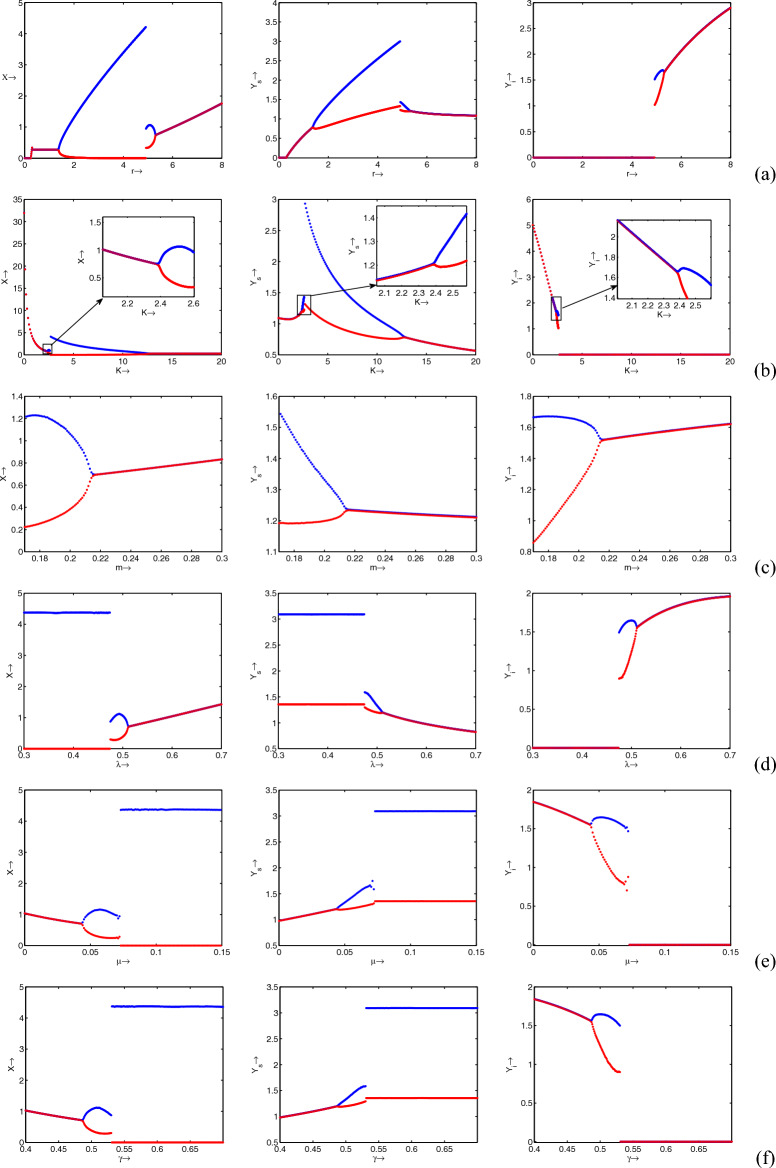
Bifurcation diagrams of system (1) with respect to the parameters (**a**) *r*, (**b**) *K*, (**c**) *m*, (**d**) $\lambda$, (**e**) $\mu$ and (**f**) $\gamma$. Rest of the parameters are at the same values as in Table 1.

Now, we see the effect of important model parameters on the system’s dynamics by varying those parameters in an interval of values. It is clear from Fig. 6a that for lower values of *r*, the system exhibits a disease-free stable state up to a certain value after that system oscillates around the disease-free state. Note that in this case fluctuation level is very high. After that disease affected the system and the system shows oscillatory behavior around the endemic state. Here the level of fluctuation is very low. Further higher values of *r* give a stable endemic state. It is apparent from Fig. 6b, that opposite dynamics could be observed for the parameter *K* with respect to the parameter *r*. That is as the value of *K* changing system exhibits a stable endemic state to an oscillating disease-free state to a stable disease-free state. The refuge parameter stabilizes the endemic state from the oscillatory state, Fig. 6c. An oscillatory disease-free state with higher amplitude is observed for lower values of $\lambda$ and moderate values of $\lambda$, system exhibits an endemic state with oscillating behavior of lower amplitude. Finally, the system falls into the stable endemic state for higher values of $\lambda$, Fig. 6d. Clearly, a totally opposite dynamics can be seen for the parameters $\mu$ and $\gamma$ to the parameter $\lambda$ (see Fig. 6e,f). Therefore, one can easily conclude from these bifurcation results that by manipulating these parameters, we can easily remove the disease from the system.

**Figure 7:**
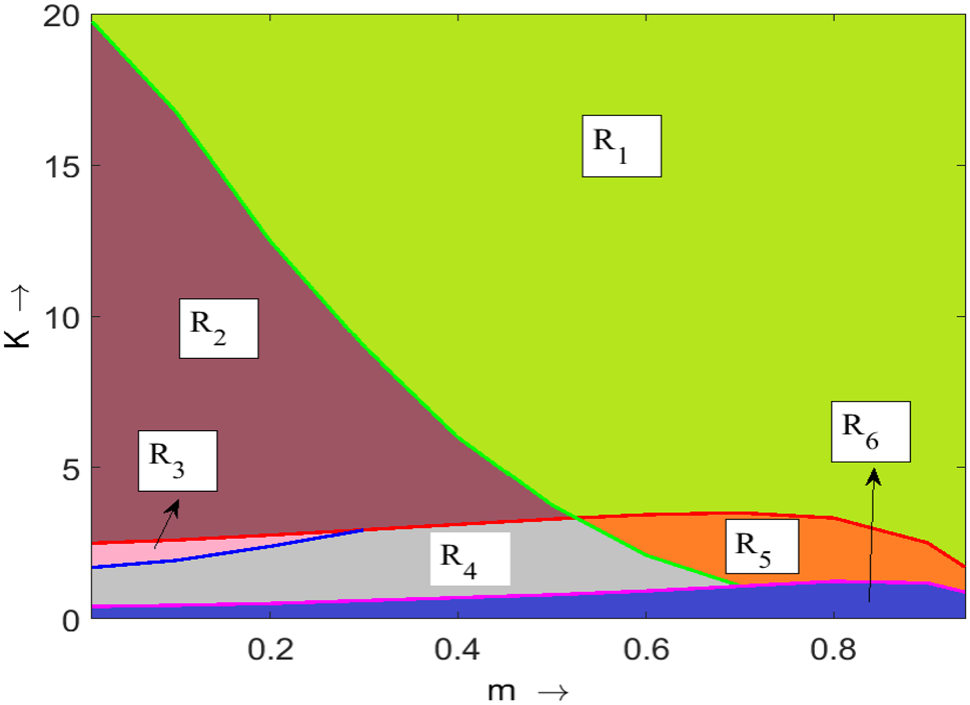
Two parametric bifurcation diagram with respect to *m* and *K*. Remaining parameters have the same values as in Table 1.

**Table 2:** Equilibrium points and their characteristics in each region as illustrated in Fig. 7.

Region	Equilibrium point	Characteristic of equilibrium point
$R_1$	$E_0, \ {\widehat{E}}, \ {\overline{E}}$	Saddle, saddle, stable
$R_2$	$E_0, \ {\widehat{E}}, \ {\overline{E}}$	Saddle, saddle, unstable spiral (stable limit cycle)
$R_3$	$E_0, \ {\widehat{E}}, \ {\overline{E}}, \ {\widetilde{E}}_1, \ {\widetilde{E}}_2$	Saddle, saddle, unstable spiral (stable limit cycle), unstable, unstable spiral (stable limit cycle)
$R_4$	$E_0, \ {\widehat{E}}, \ {\overline{E}}, \ {\widetilde{E}}_1, \ {\widetilde{E}}_2$	Saddle, saddle, unstable spiral (stable limit cycle), unstable, stable
$R_5$	$E_0, \ {\widehat{E}}, \ {\overline{E}}, \ {\widetilde{E}}_1, \ {\widetilde{E}}_2$	Saddle, saddle, stable, unstable, stable
$R_6$	$E_0, \ {\widehat{E}}, \ {\overline{E}}, \ {\widetilde{E}}$	Saddle, saddle, unstable, stable

**Figure 8:**
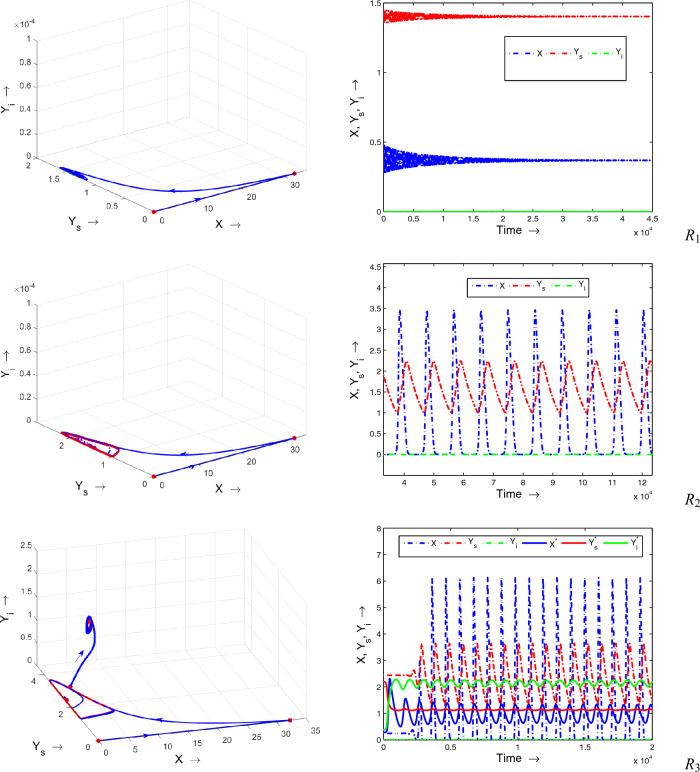
The complete phase portrait along with the corresponding time series for the various regions outlined in Fig. 7 are presented for the system (1). Remaining parameters adopted from Table 1.

**Figure 9:**
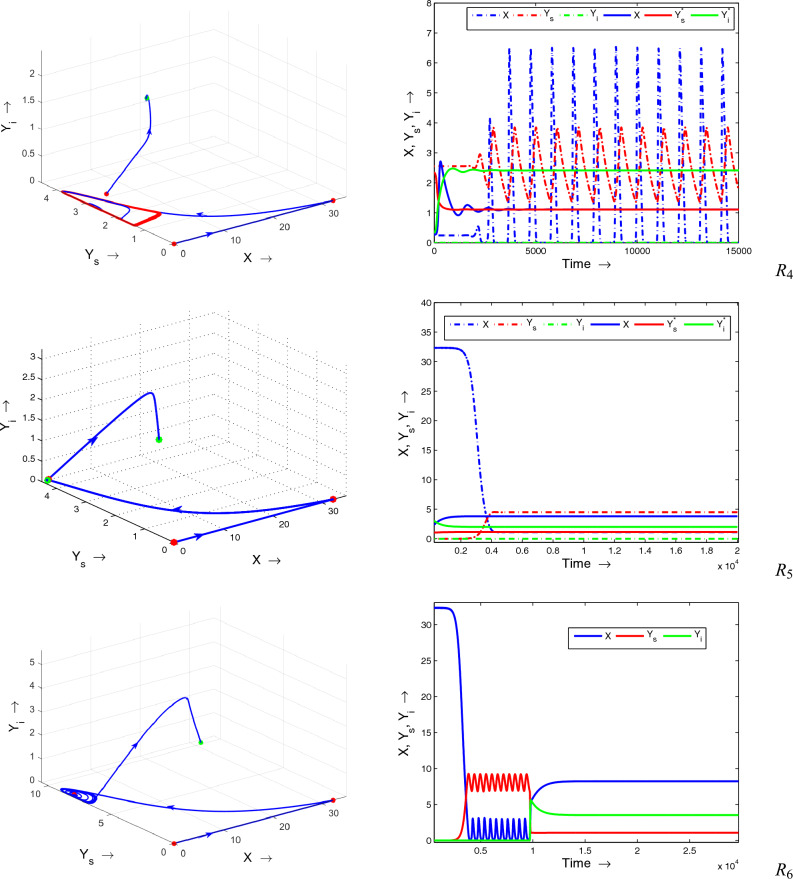
The complete phase portrait along with the corresponding time series for the various regions outlined in Fig. 7 are presented for the system (1). Remaining parameters adopted from Table 1.

Next, to see the effect of fear and refuge on the system’s dynamics, we draw two parametric bifurcation diagram by varying these parameters (*m* and *K*) at once. The whole region is divided into 6 subregions ($R_1$ $-$ $R_6$) with different dynamics, Fig. 7. It is clear from the figure that there is a large region $R_1$ in which a disease-free state is stable (see Fig. 8$R_1$), which is biologically most preferable region. In region $R_2$, disease-free equilibrium oscillates, Fig. 8$R_2$. Note that in these two regions diseases can not persist in the system. It indicates that higher values of both fear and refuge give a disease-free state. Next, as the system enters into region $R_3$, it shows bistability, i.e., here our system shows oscillatory behavior around both endemic and disease-free equilibrium (see Fig. 8$R_3$). That is in $R_3$ whether disease will persist or not persist, depends on the initial population size. Further, for lower values of *K*, the system enters into the region $R_4$. In this case, also bistability occurs. But, here endemic equilibrium is stable and disease-free equilibrium behaves periodically, Fig. 9$R_4$. It is apparent from Fig. 9$R_5$ that in region $R_5$ both disease-free and endemic states are stable. Finally, in region $R_6$, interesting dynamics can be observed. Here, up to a certain time system behaves in an oscillatory manner around the disease-free state and ultimately goes to the endemic stable state (see Fig. 9$R_6$). Furthermore, all the characteristics of each subregions (($R_1$ $-$ $R_6$)) are explicitly given in Table 2. One can easily follow this table by corresponding region’s figures.

**Figure 10:**
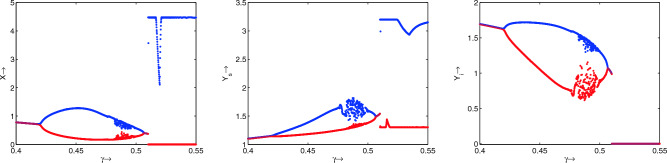
Bifurcation diagrams of system (1) with respect to the treatment parameter $\gamma$. Rest of the parameters are at the same values as in Table 1 except $\mu = 0.075$.

**Figure 11:**
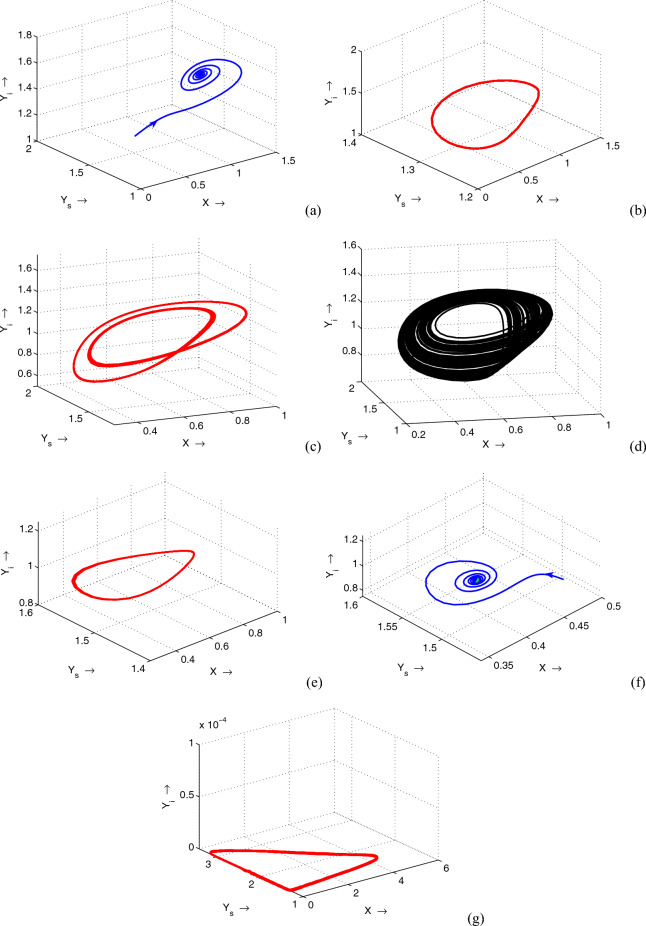
Phase portraits of system (1) as a justification of the bifurcation diagram, Fig. 10. Parameters are same as in Fig. 10 except in (**a**) $\gamma =0.41$, (**b**) $\gamma =0.45$, (**c**) $\gamma =0.48$, (**d**) $\gamma =0.49$, (**e**) $\gamma =0.50$, (**f**) $\gamma =0.508$ and (**g**) $\gamma =0.52$.

Moreover, for a different value of $\mu$ other than the value mentioned in Table 1, we plot the bifurcation diagram with respect to the treatment parameter $\gamma$, Fig. 10. All the bifurcating results due to the variance of $\gamma$ are depicted in phase portraits plotted in Fig. 11. It is transparent from these phase portraits that for lower values of $\gamma$, the system shows a stable endemic state. As the value of $\gamma$ increases, the system exhibits one period to two periods to chaos to one period to a stable state of endemic equilibrium. Finally, for higher values of $\gamma$, the system settles to a periodic disease-free state.

**Figure 12:**
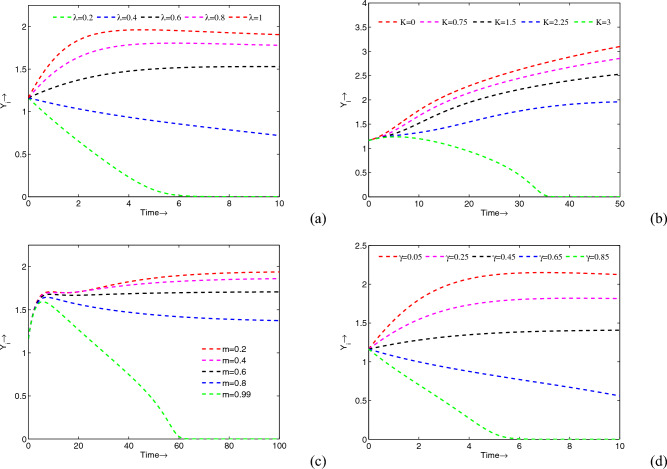
Variations in the infected predator populations for different values of (**a**) $\lambda$, (**b**) *K*, (**c**) *m* and (**d**) $\gamma$. Parameters are at the same values as in Table 1 except in (**b**) $\gamma =0.51$ (**c**) $\gamma =0.31$.

Furthermore, to be more transparent about the change of density of the infected population by varying model parameters $\lambda$, *K*, *m* and $\gamma$ see Fig. 12. It is apparent from the figures that $\lambda$ is directly proportional to the density of the infected population whereas other parameters (*K*, *m* and $\gamma$) are inversely proportional to infected population density. It means if the value of $\lambda$ increases (decreases), the density of the infected population increases (decreases) accordingly. On the other hand, if the value of each of these parameters *K*, *m*, and $\gamma$ increases (decreases), the density of the infected population decreases (increases) accordingly. Thus, from these results also, one can conclude that the infection can be suppressed by fear, refuge, and treatment. From the biological point of view, these results are very important to conserving any ecosystem.

**Figure 13:**
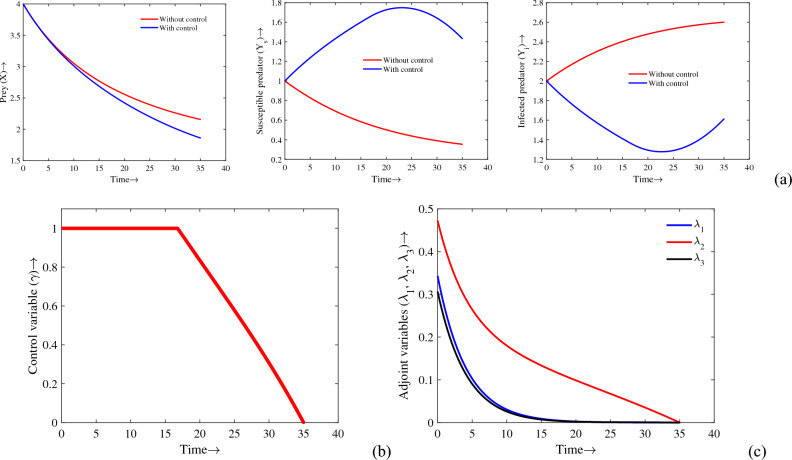
Time series solutions of (**a**) each population with and without control; (**b**) treatment control ($\gamma$); (**c**) adjoint variables ($\lambda _i, \ i=1,2,3$). Parameters are at the same values as in Table 1. Moreover, we take $A=0.01$ and $B=0.05$.

In order to solve the optimality system numerically, the forward-backward sweep method^54^ has been used. Some initial guesses about the control variable are made before the process begins. Runge–Kutta fourth order iterative scheme is used first to solve the system of state variables forward in time, then to solve the system of adjoint variables backward in time. In each iteration, the control value is updated. Until the desired convergence is achieved, the process is repeated. Weight factors associated with infected predator and treatment are chosen as $A=0.01$ and $B=0.05$, whereas the other parameter values are same as in Table 1 with the initial population sizes $X(0)=4$, $Y_s=1$ and $Y_i=2$. Moreover, we consider the time interval as [0, 35]. The simulations for the density of prey, susceptible predator, and most important infected predator populations under the optimal control parameter $\gamma (t)$ and without control strategies are shown in Fig. 13. The drop in the infected predator density and rise in the susceptible predator density in presence of time-dependent optimal control is apparent in the figure. A plot of $\gamma (t)$’s profile is shown in Fig. 13b,c along with the transversality condition $\lambda _i(t)=0$. In this figure, it can be seen that it is optimal to exert efforts until 16.7 units of time, then lower them down. As time increases, $\lambda _i$ converges to zero as we observe in the adjoint variables. From these figures, one can say that optimal control in treatment is very effective to reduce the number of infected predators and eradicate the infection from the system.

## Discussion and conclusion

This research proposes a novel predator–prey model incorporating the effects of fear and fear-induced refuge, where the predator population can become infected by pathogens or viruses. The study introduces a treatment strategy for the infected predator individuals. To the best of our knowledge, this type of predator–prey model involving treatment is innovative and previously unexplored. The primary objective of this study is to investigate the impacts of fear, fear-induced refuge, and treatment on an infected predator–prey system. To validate the model, we first analyze the positivity and boundedness of the system’s solutions. Subsequently, we examine the biologically feasible equilibria of the system. Interestingly, we derive a reproduction number that plays a crucial role in discussing the possibilities of disease elimination and persistence within the system. This analysis involves rigorous exploration of Backward bifurcation, as well as local and global stability. Additionally, we delve into the dynamics of the system by studying Saddle-node and Hopf bifurcations. In addition to exploring the system’s dynamic behavior, we also delve into the dynamics of the infected population. Here, our goal is to mitigate the density of the infected population by exerting control over infected individuals and the medical resources allocated for treatment. To this end, we establish the framework of an optimal control problem and leverage Pontryagin’s maximum principle to arrive at a solution. Optimal control emerges as significantly more potent in diminishing the density of the infected population while concurrently augmenting the density of susceptible predators.

Our findings suggest that when the basic reproduction number, denoted as $R_0$, is less than a critical threshold $R_0^*$, the disease is unable to sustain itself within the system. However, within the range $R_0 \in [R_0^*, 1]$, the persistence of the disease becomes contingent upon the initial population sizes. Conversely, once $R_0$ surpasses unity, the disease becomes a permanent fixture within the system. Furthermore, our bifurcation analyses indicate that the individual growth rate of prey and the disease prevalence rate independently contribute to the escalation of the infected population’s density. In contrast, factors such as fear, fear-induced refuge, recovery rate, and treatment each play a distinct role in diminishing the density of the infected population. Remarkably, higher values of these influencing factors ultimately lead to the eradication of infected individuals from the system. Moreover, our findings indicate that when fear is at a very low level, the infected population persists within the system for all future time intervals. Increasing the level of fear introduces a nuanced dynamic: the persistence or extinction of the disease depends on the initial population size. Nevertheless, it’s important to note that the infected population cannot sustain itself within the system when fear and refuge are elevated to higher levels.

Furthermore, through manipulation of the recovery rate, intriguing dynamics emerge. As the treatment intensity increases, the system transitions from a single periodic behavior to two periodic cycles, further evolving into multiple periodic or even chaotic states, and then reverts back to a single periodic pattern. Ultimately, the system stabilizes into an endemic state before eventually converging to a disease-free periodic state. This exploration unveils an interesting phenomenon: when medical resources are limited, the disease persists, accompanied by chaotic behavior. Conversely, an ample supply of medical resources can effectively manage the chaos and lead to disease eradication. The overarching conclusion drawn from this investigation is that the study’s findings hold significant real-world implications. Moreover, it sheds fresh light on the intricate dynamics inherent in a predator–prey system afflicted by infections within the predator population.

The proposed model finds relevance in diverse biological scenarios where predator–prey dynamics and disease interactions are influenced by ecological factors such as prey refuge and fear-induced behaviors. The model can be applied in fields like wildlife conservation, agricultural systems, epidemiology, disease ecology, ecological restoration, and behavioral ecology. The adaptability of the model to simulate different scenarios underscores its capacity to address a wide range of ecological contexts where predator-prey dynamics and disease interactions play a pivotal role.

In wildlife conservation setting, where the predators could represent natural predators like wolves or big cats, and the prey could be animals like ungulates or small mammals. The inclusion of disease dynamics and ecological effects like refuge and fear could provide insights into disease spread within wildlife populations and how these interactions impact conservation efforts. Agricultural Systems: In agricultural settings, the predator–prey dynamics could be relevant to pests and their natural predators. Understanding how disease transmission and fear-induced behaviors impact the dynamics of pest control can have implications for sustainable agriculture practices.

## Data Availability

All data generated or analyzed during this study are included in this article. The softwares used in this study are Maple (version—2019) and MATLAB (version—R2019a).
